# Emotion Recognition Based on Fusion of Topological Features and Trajectory Images Derived from EEG Phase Space Reconstruction

**DOI:** 10.3390/s26103102

**Published:** 2026-05-14

**Authors:** Tianyue Liang, Xuanpeng Zhu, Yu Song

**Affiliations:** 1 Beijing-Dublin International College, Beijing University of Technology, Beijing 100124, China; 2 School of Electrical Engineering and Automation, Tianjin University of Technology, Tianjin 300384, China

**Keywords:** phase space reconstruction, topological features, trajectory images, EEG emotion recognition

## Abstract

Electroencephalogram (EEG) signals, as a direct measure of the brain’s cortical electrophysiological activity, can objectively capture emotion-induced neural changes. Phase space reconstruction is an effective method for processing nonlinear time series. It maps time series to a high-dimensional phase space, thereby better preserving subtle dynamic information in the signal. This paper proposes a method for emotion recognition in EEG signals based on phase space reconstruction. First, the macro-topological features of the trajectories are constructed via phase space reconstruction. The time delay and embedding dimension are then optimized using the minimum cross-prediction error and the G-P method, followed by dimensionality reduction to a two-dimensional plane via local linear embedding. Building on this foundation, and in response to the limitations of manually designed features, we further propose a deep learning-based method for extracting multiscale dynamic features from trajectory images. The designed GN-MVXXS framework, which utilizes a granularity-adaptive module to adaptively switch the receptive field and a noise-filtering module to suppress isolated noise points, thereby effectively uncovers microscopic evolutionary features at the image level. Finally, to leverage the complementary strengths of macro- and micro-level information, we propose a fusion method based on dynamic attention. This approach aligns the dual representational dimensions through global average pooling and nonlinear dimension expansion, and utilizes a dynamic attention mechanism to adaptively assign feature weights, enabling the model to collaboratively enhance both overall dynamic patterns and local details based on sample characteristics. The experimental results show that the model achieved an accuracy of 96.11% in the three-class classification task on the SEED, 86.33% in the four-class classification task on the HIED, and 83.67% in classification across normal-hearing and hearing-impaired individuals, significantly outperforming single-feature models and traditional fusion methods.

## 1. Introduction

Emotions are a complex state of mind; they reflect an individual’s attitude toward the relationship between their own needs and the external world, and are an integral part of daily life [1]. With the advancement of artificial intelligence, emotion recognition has found widespread application and has become one of the key technologies for enhancing the intelligence and naturalness of human–computer interaction [2]. Sentiment recognition is typically evaluated using sentiment models. Depending on how sentiment is expressed, the sentiment models currently in widespread use are primarily divided into two types: discrete and continuous [3]. Discrete emotion models view emotions as independent, discrete states, positing that emotions consist of several basic emotions, with other complex emotions arising from different combinations of these basic emotions. Dimensional emotion models, on the other hand, construct a continuous emotional space to describe a range of complex, nuanced, and continuous emotional states [4].

Based on the type of signal, emotion recognition methods can be broadly categorized into two main types: those based on non-physiological signals and those based on physiological signals. Unlike non-physiological signals, which can be easily masked, physiological signals directly reflect internal physiological activity and are not influenced by an individual’s cognition or subjective intent, thereby offering greater objectivity [5]. As one type of non-physiological signal, electroencephalogram (EEG) signals have received widespread attention and application in emotion recognition research [6].

Research on emotion recognition using EEG signals primarily involves experimental paradigms, data preprocessing, feature extraction, and emotion classification [7]. Feature extraction plays a crucial role in EEG-based emotion recognition and has therefore attracted widespread attention. Given the complex nonlinear characteristics of EEG signals, some researchers have focused their feature extraction studies on the application of nonlinear analysis methods [8]. Yuvaraj et al. [9] extracted features such as sample entropy and approximate entropy from EEG signals to evaluate the performance of Parkinson’s patients across six different emotional states. Tuncer et al. [10] proposed a feature extraction method based on fractal patterns and used support vector machines to perform a four-class sentiment recognition task. Yang et al. [11] utilized nonlinear features such as Lyapunov exponents and fractal dimensions to validate the model performance of a four-class emotion experiment conducted with independent participants. In addition to these nonlinear features, entropy-based measures such as spectral entropy, approximate entropy, sample entropy, and phase entropy have also been widely applied in EEG analysis [12]. Previous studies have primarily focused on the quantitative analysis of the local dynamical properties of EEG signals, but have lacked a systematic characterization of the global evolution of dynamical systems. In this paper, we use phase space reconstruction to transform EEG signals into continuous trajectories, thereby constructing topological features. By combining geometric and dynamical perspectives, we comprehensively capture emotion-related nonlinear patterns.

Phase space reconstruction was first applied to the analysis of real-world time series in complex systems. In their study of time series in complex systems, Small et al. [13] applied phase space reconstruction to the analysis of real observational data, using reconstructed phase space trajectories to characterize the system’s nonlinear dynamical behavior. They validated the effectiveness of this method in practical applications through physical experimental systems and chaotic time series data. Marwan et al. [14] combined phase space reconstruction with recursive analysis methods and applied them to the analysis of climate and geophysical time series. By revealing the dynamic evolutionary characteristics of the system through changes in phase space structure, they advanced the application of this method in complex natural systems. Yan et al. [15] introduced phase space reconstruction into the analysis of mechanical vibration signals and equipment health monitoring; by analyzing the reconstructed phase space trajectories and their nonlinear characteristics, they achieved effective differentiation between various operating states and failure modes. The above studies demonstrate that phase space reconstruction is well-suited for a wide range of real-world systems and provides an effective method for the dynamic analysis and state identification of complex signals.

With the successful application of phase space reconstruction methods in the analysis of real-world complex systems, researchers have gradually begun to apply them to the analysis of electroencephalographic signals, which exhibit significant nonlinearity and nonstationarity [16]. When using EEG to localize epileptic foci, Zeng et al. [17] employed empirical mode decomposition to obtain eigenmodes, extracted the third and fourth eigenmode components—which account for most of the EEG’s energy—and reconstructed the phase space of these two components, within which they calculated Euclidean distance as a feature. Yan et al. [18] extracted multi-band continuous homogeneous features from the EEG phase space for use in emotion classification tasks. Pourali et al. [19] fit Poincaré sections in phase space to analyze data trajectories. They used evolutionary algorithms to learn the hyperparameters for phase space reconstruction and Poincaré sections, employing statistical features extracted from Poincaré intersections to classify left/right-hand and left/right-foot movements. Yao et al. [20] used phase space reconstruction techniques to capture the nonlinear dynamical characteristics of EEG signals and combined this with complex network analysis to extract features for distinguishing between sad, neutral, and happy emotions. The studies have demonstrated the feasibility of phase space reconstruction in the nonlinear dynamic analysis of EEG signals, providing valuable insights for this research; however, most methods still rely on feature extraction from a single perspective. How to systematically extract emotional information from phase space trajectories at both the macro-topological and micro-trajectory levels remains to be further explored. This paper converts EEG signals into continuous trajectories through phase space reconstruction, thereby constructing topological features. By integrating geometric and dynamical perspectives, it comprehensively captures emotion-related nonlinear patterns. Crucially, both the macro-level topological features and the micro-level trajectory images originate from the same phase space reconstruction of identical EEG segments, enabling a physically homogeneous dual representation. We systematically extract emotional information from phase space trajectories at these two complementary levels. The specific work is as follows:

1. We have developed a method for constructing topological features based on phase space reconstruction, which characterizes the overall dynamic properties of trajectories at the macro level. By employing the MCPE and GP algorithms to optimize time delay and embedding dimension, respectively, we achieve high-quality phase space reconstruction. Through LLE dimension reduction, we mapped high-dimensional trajectories onto a two-dimensional plane while preserving the original manifold structure, and constructed topological features to overcome the inherent limitations of traditional linear methods.
2. To address the multi-scale distribution and noise interference inherent in trajectory images, we developed an improved model, GN-MVXXS, based on the MobileViT-XXS architecture by introducing two modules: granularity adaptation (GA) and noise filtering (NF). The GA module dynamically adjusts the feature extraction receptive field based on trajectory density, while the NF module effectively suppresses spatially isolated noise. Together, these modules address the limitations of traditional models, which suffer from a single-feature extraction scale and susceptibility to noise interference.
3. We propose a dual-representation fusion strategy based on dynamic attention. A two-layer fully connected network was used to perform nonlinear mapping of topological features, thereby achieving dimensional unification and precise alignment of the dual representations. Subsequently, a dynamic attention fusion mechanism is introduced. By calculating the interactive correlations between features to adaptively assign representation weights, the model can dynamically balance the contributions of macro-structure and micro-details based on sample characteristics, thereby overcoming the limitations of single-representation approaches.

## 2. Dataset

The SEED is one of the most used public datasets in the field of EEG-based emotion recognition [21]. The dataset comprises 15 healthy participants (seven males and eight females) and includes 15 movie clips representing three emotions: sadness, neutral, and happiness. Each clip is approximately 4 min long.

The Hearing Impaired EEG Emotion Dataset (HIED) comprises 20 film clips representing four emotions (sadness, neutral, happiness, and fear) [22]. The total number of participants was 15, comprising 12 males and three females, all from the School of Deaf Education at Tianjin University of Technology. Their average age was 22 years. All participants were native Chinese speakers who primarily used sign language for daily communication and wore hearing aids. The EEG signals obtained from the experiment contain a significant amount of noise, including electrooculogram (EOG), electromyogram (EMG), and power-line interference. Therefore, preprocessing is required to minimize these effects as much as possible.

To reduce the complexity of subsequent calculations, the sampling rate of the EEG data was downsampled from 1000 Hz to 200 Hz. A bandpass filter with a passband of 1–75 Hz was applied to remove high- and low-frequency noise, and a notch filter centered at 50 Hz was used to eliminate power-line interference. Using Independent Component Analysis (ICA), the EEG data were decomposed into independent components. An automated classification procedure identified components likely to represent brain activity; non-brain components were removed as artifacts. The remaining components were then back-projected to reconstruct artifact-corrected EEG signals. Cheema et al. reported that denoising prior to phase space reconstruction improves nonlinear dynamic characterization, supporting our preprocessing approach [23].

To ensure the validity and consistency of the analysis and to avoid external factors such as subject fatigue and distraction from affecting the results, the EEG data from the final 180 s of each movie clip for each subject were selected for subsequent feature extraction and image generation. To fully characterize the differences in phase space trajectories and ensure an adequate sample size, the 180 s data were segmented using non-overlapping 9 s time windows, with each window treated as an independent sample [24]. Consequently, 20 independent samples were obtained for each participant for a single movie clip.

## 3. Feature Extraction

Since the present work extends our previous study [25], the parameter determination and the extraction of partial topological features in this section follow the same procedures as described in [25] to maintain methodological consistency.

### 3.1. Topological Features

Regarding the process of phase space reconstruction, we first assume that the original EEG signal is a one-dimensional discrete-time sequence ${\left\{s_{n}\right\}}_{n=1}^{N}$, where $s_{n}$ represents the EEG signal amplitude at the n-th sampling time point, and N is the sequence length n∈{1,2,…,N}.

**Figure 1:**
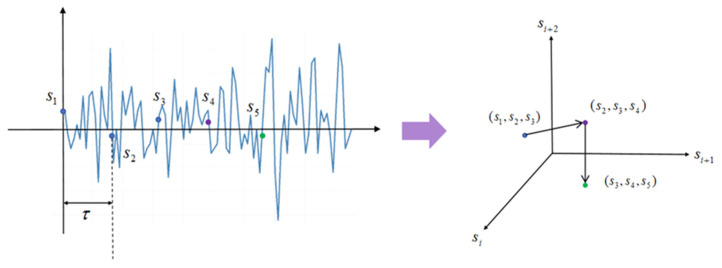
Schematic diagram of the reconstruction process.

Based on this, assuming that $s_{n}$ is the n-th point in the original EEG sequence, the point in phase space is reconstructed as follows:

(1) $$P_{n}=(s_{n},s_{n+\tau },\ldots ,s_{n+(d-1)\tau })$$

where $P_{n}$ represents the n-th point in the reconstructed phase space. Let τ denote the time delay and d denote the embedding dimension. Time delay τ is measured in discrete sampling points and represents the sampling interval between adjacent reconstructed components (if the sampling frequency is $f_{s}$, the corresponding actual time delay is $\frac{\tau }{f_{s}}$). Figure 1 illustrates the reconstruction process.

In phase space reconstruction, the choice of time delay τ and embedding dimension d directly affects the quality of the reconstruction and the system’s ability to resolve dynamics. This paper employs the Minimum Cross Prediction Error (MCPE) method to determine the time delay τ. It minimizes prediction errors based on a polynomial regression model, enabling it to adaptively capture complex dynamics and suppress noise interference. The Grassberger–Procaccia (G-P) method is used to compute the embedding dimension d, and the geometric properties of the trajectories are dynamically quantified based on the proximity density of point pairs in phase space to preserve the system’s topological structure.

The core idea of MCPE is to determine time delays by predicting future values of a time series and evaluating their correlation with lagged versions [26]. The specific process is as follows:

First, define the original time series $T=(t_{1},t_{2},t_{3},\ldots ,t_{n})$. For different time delay parameters, perform a cyclic shift on the original data to obtain series $Z=(z_{1},z_{2},z_{3},\ldots ,z_{n})$, thereby simulating the effect of time delay.

Next, the data are fitted using quadratic polynomial regression, with the following equation:

(2) $$T=q_{0}+q_{1}Z+q_{2}Z^{2}$$

where represents the constant term, the first-order coefficient, and the second-order coefficient, respectively. Using regression analysis, estimate the optimal parameter $\hat{q_{0}},\hat{q_{1}},\hat{q_{2}}$ and substitute the coefficient into the right-hand side of the formula to calculate the predicted sequence $T_{pred}$. Next, calculate the mean squared error between the predicted and actual values:

(3) $$T_{pred}=\hat{q_{0}}+\hat{q_{1}}Z+\hat{q_{2}}Z^{2}$$

Finally, compare the mean squared error corresponding to different time delays:

(4) $$\mathrm{MSE}=\frac{1}{n}\sum_{i=1}^{n}(T_{i}-T_{pred,i}{)}^{2}$$

**Figure 2:**
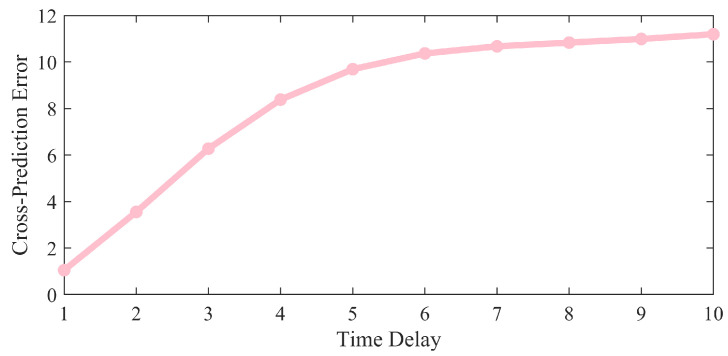
Mean squared error at different time delays.

As shown in Figure 2, the MSE from A to B was calculated in this study. The results show that the MSE reaches its minimum at time τ=1, indicating the strongest correlation among the variables; based on this, the value of τ is set to one.

The G-P method aims to determine the minimum embedding dimension that accurately reflects the system’s dynamic characteristics by analyzing the distance relationships between points in phase space [27]. Based on the time delays determined by the MCPE method, the phase space is progressively reconstructed across different embedding dimensions, ranging from 2D to 10D. For each dimension, reconstruct the phase space, calculate the number of point pairs $C_{t}$, and compute the distance $L_{t}$ between the two points in each pair. Next, filter out $C_{s}$ pairs of points where the distance from $L_{t}$ is less than the standard deviation of the original time series, and calculate the ratio:

(5) $$Q=\frac{C_{s}}{C_{t}}$$

**Figure 3:**
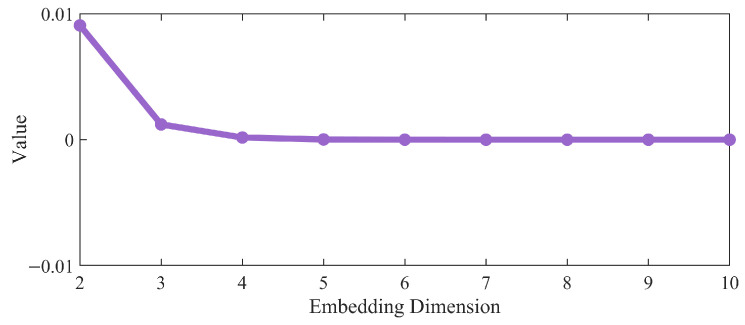
Values at different embedding dimensions.

As shown in Figure 3, by comparing the values of Q across different embedding dimensions, we observe that the value of Q gradually stabilizes starting from the three-dimensional case. This indicates that three-dimensional phase space can effectively characterize the system’s dynamic properties; therefore, d=3 is selected.

Reducing high-dimensional trajectories to a two-dimensional plane facilitates better visualization and further feature extraction. At the same time, it is essential to preserve important similarities and relationships while maintaining the original high-dimensional local structure. This study employs the local linear embedding (LLE) method for dimensionality reduction. By assuming that the data is linear within local neighborhoods and preserving the relative positions of data points among their local neighbors, this method effectively retains the local structure of the data [28].

For each point $V_{1},V_{2},\ldots ,V_{n}$ in a high-dimensional space, calculate its Euclidean distance from all other points in the vicinity, and select the p points closest to it as its neighbors, denoted by $\left\{V_{i_{1}},V_{i_{2}},\ldots ,V_{i_{p}}\right\}$.

Next, the local linear weight matrix W is determined by minimizing the reconstruction error. Assuming that each sample point can be linearly reconstructed from its neighbors, the weight matrix $W_{ij}$ represents the contribution of point $V_{i}$ to its j-th neighbor. The objective for minimizing the reconstruction error is defined as follows:

(6) $$\mathrm{RE}=\min{\sum_{i=1}^{n}\left||V_{i}-\sum_{j=1}^{p}W_{ij}V_{i_{j}}|\right|}^{2}$$

The sum of the weights for each sample point must equal one; the sparse weight matrix W is obtained by solving this equation.

During the low-dimensional embedding stage, a symmetric matrix is constructed using the weight matrix, with the following expression:

(7) $$M={\left(I-W\right)}^{T}(I-W)$$

where I is the identity matrix.

Finally, perform an eigenvalue decomposition on matrix M:

(8) Mμ=λμ

Select the eigenvectors $\mu_{2},\mu_{3}$ corresponding to the two smallest eigenvalues (excluding the smallest eigenvalue $\mu_{1}$), and permute them to generate a low-dimensional embedding U:

(9) $$U={\left[\mu_{2},\mu_{3}\right]}^{T}$$

In LLE, the choice of the number of nearest neighbors p has a significant impact on the dimension reduction performance. Based on the principle of minimizing reconstruction error described earlier, the optimal value of p must be determined through systematic parameter selection.

**Figure 4:**
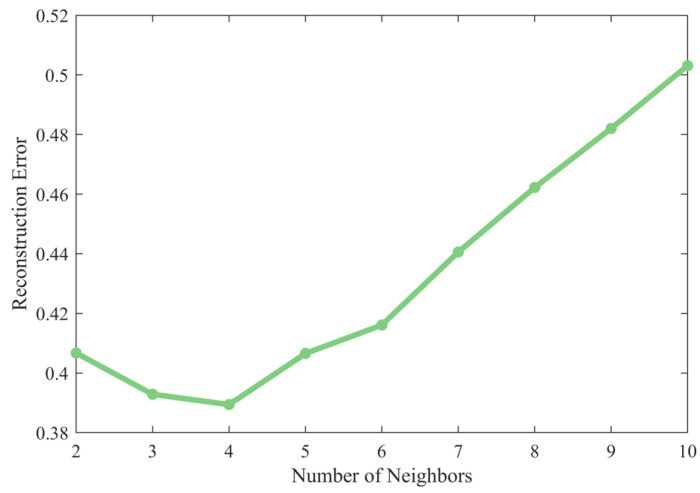
Reconstruction error for different numbers of nearest neighbors.

By calculating and analyzing the reconstruction error RE from p=2 to p=10 (since the number of nearest neighbors must be no less than the dimension after dimensionality reduction, p=1 does not exist), as shown in Figure 4, the reconstruction error reaches its global minimum when p=4. Since this model is able to fully capture the continuity of the manifold’s local structure while avoiding the violation of the linearity assumption caused by an overly large neighborhood, model p=4 was ultimately selected.

**Figure 5:**
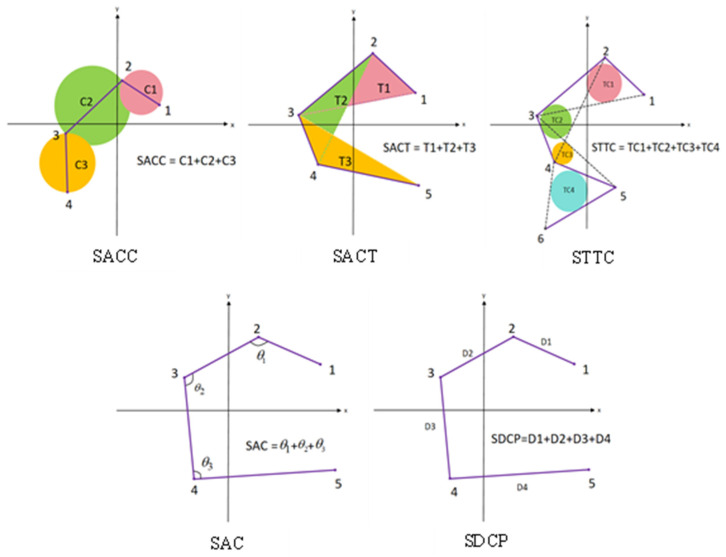
Schematic diagram of 5 topological features.

After reducing the dimensionality of the high-dimensional trajectories using LLE, the five topological features used here were filtered from a larger set in our prior work [25] and are defined as follows. A schematic diagram of all features is shown in Figure 5.

Any two consecutive points form a vector; consider the length of this vector to be the diameter of a circle. Find the area of this circle, and calculate the sum of the areas of all such circles.

(10) $$\mathrm{SACC}=\sum_{i=1}^{N-1}\frac{\pi }{4}[{\left(X_{i+1}-X_{i}\right)}^{2}+{\left(Y_{i+1}-Y_{i}\right)}^{2}]$$

where N represents the number of points in a two-dimensional plane, $X_{i}$ and $Y_{i}$ represent the x- and y-coordinates of the points, respectively, and i represents the sequence number of consecutive points.

For every three consecutive points that form a triangle, find the area of that triangle and calculate the sum of the areas of all the triangles (SACT).

(11) $$\mathrm{SACT}=\frac{1}{2}\sum_{i=1}^{N-2}\left|\det\left[\begin{array}{ccc} X_{i} & Y_{i} & 1\\ X_{i+1} & Y_{i+1} & 1\\ X_{i+2} & Y_{i+2} & 1 \end{array}\right]\right|$$

Every three consecutive points form a triangle. Find the incircle of all the triangles and calculate the sum of the areas (STTC). The three sides a, b, and c of the triangle are calculated as follows

(12) $$a=\sqrt{{\left(X_{i+1}-X_{i}\right)}^{2}+{\left(Y_{i+1}-Y_{i}\right)}^{2}}$$

(13) $$b=\sqrt{{\left(X_{i+2}-X_{i+1}\right)}^{2}+{\left(Y_{i+2}-Y_{i+1}\right)}^{2}}$$

(14) $$c=\sqrt{{\left(X_{i+2}-X_{i}\right)}^{2}+{\left(Y_{i+2}-Y_{i}\right)}^{2}}$$

The area of a triangle is calculated as

(15) $$S=\sqrt{\frac{a+b+c}{2}\times \frac{b+c-a}{2}\times \frac{a+c-b}{2}\times \frac{a+b-c}{2}}$$

Using S to find the radius of its inscribed circle r is

(16) $$r=\frac{2S}{a+b+c}$$

The final STTC calculation results are as follows:

(17) $$\mathrm{STTC}=\sum_{j=1}^{N-2}\pi r_{j}^{2}$$

where j is the index of the incircle of the triangle.

A vector is formed between any two consecutive points, and an angle is formed between any two consecutive vectors. Calculate the sum of the measures of all these angles (SAC).

(18) $$\mathrm{SAC}=\sum_{i=1}^{N-2}\arccos\frac{\left(X_{i+1}-X_{i})(X_{i+2}-X_{i+1})+(Y_{i+1}-Y_{i})(Y_{i+2}-Y_{i+1}\right)}{\sqrt{{\left(X_{i+1}-X_{i}\right)}^{2}+{\left(Y_{i+1}-Y_{i}\right)}^{2}}+\sqrt{{\left(X_{i+2}-X_{i+1}\right)}^{2}+{\left(Y_{i+2}-Y_{i+1}\right)}^{2}}}$$

Any two consecutive points form a vector; calculate the sum of the lengths of all vectors (SDCP).

(19) $$\mathrm{SDCP}=\sum_{i=1}^{N-1}\sqrt{{\left(X_{i+1}-X_{1}\right)}^{2}+{\left(Y_{i+1}-Y_{1}\right)}^{2}}$$

### 3.2. Trajectory Images

**Figure 6:**
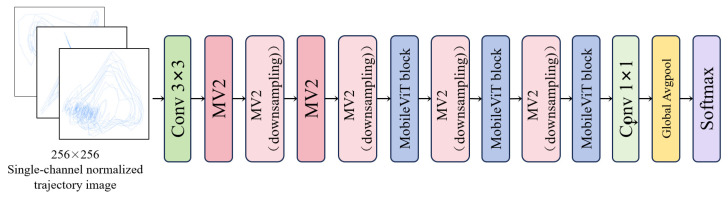
Overall architecture of MobileViT-XXS.

Topological features rely on statistics derived from manually designed prior models, making it difficult to fully preserve the original pixel-level morphological information in trajectories. These microscopic dynamic details often contain nonlinear features closely related to emotional states, which may be lost during the quantification process. In contrast, two-dimensional trajectory images retain the complete original trajectory morphology. We propose the GN-MVXXS model, an improved version of MobileViT-XXS (as Figure 6), for deep feature extraction and classification of trajectory images after dimension reduction via LLE.

Trajectory images often suffer from issues such as inconsistent dimensions and variations in pixel distribution; feeding them directly into a model can lead to unstable training and poor generalization. To ensure image consistency and trajectory integrity, the dimensions of the trajectory images are standardized to 256 × 256 during processing, thereby avoiding feature loss or redundant blank spaces caused by subsequent scaling or cropping. During processing, the coordinate range of the two-dimensional trajectory is dynamically calculated, and a 1% margin is added. This approach prevents the loss of trajectory details while avoiding excessive redundant blank spaces, ensuring the trajectory is complete and uniformly distributed. Next, the generated images were uniformly converted into single-channel grayscale images. Finally, pixel values were mapped from the [0,255] range to the [−1,1] range to standardize the numerical range of features and prevent dimensionality differences from affecting model training.

In the context of this study, the original MobileViT-XXS model suffers from two issues: first, its feature extraction granularity is fixed, making it unable to adapt to variations in trajectory distributions, which results in the omission of effective fine-grained features or the redundancy of coarse-grained features; second, it lacks a feature refinement mechanism; if global modeling is performed directly via a Transformer, invalid responses may appear in the channel dimension, thereby reducing feature discriminative power. To address these issues, this study introduces modifications to the MobileViT-XXS model by adding a granularity adaptation (GA) module and a noise-filtering (NF) module. The resulting model is named GN-MVXXS and is applied to trajectory image recognition and classification tasks.

**Figure 7:**
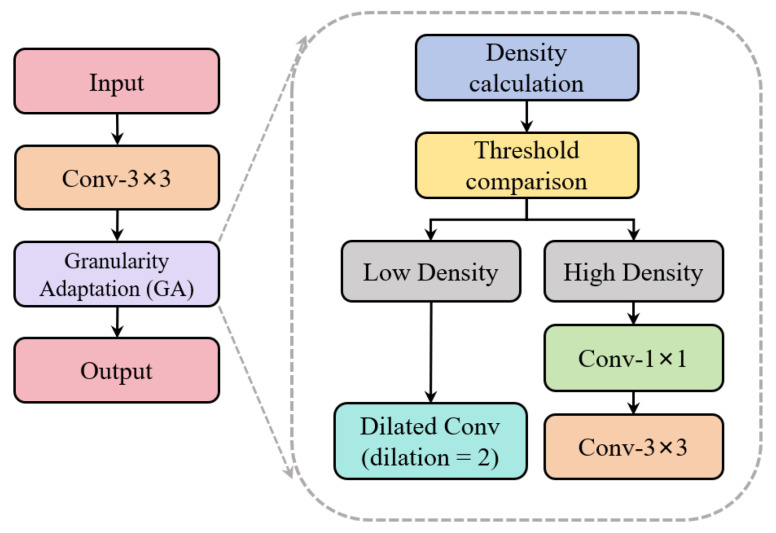
Granularity adaptation (GA) module.

The GA module is located after the initial convolutional blocks of the original model; its main structure is shown in Figure 7. At this stage, the original model uses only a standard 3 × 3 convolution kernel with fixed parameters to perform preliminary feature extraction. It lacks a pre-computation step for trajectory density and does not adjust the convolution strategy to account for differences in trajectory distribution. For low-density trajectory images, 3 × 3 convolution cannot effectively expand the receptive field to capture global distribution patterns; for high-density trajectory images, 3 × 3 convolution tends to introduce redundant computations and cannot focus on core trajectory features through channel dimension reduction.

The GA module includes a built-in submodule for calculating trajectory density. The processing workflow is as follows. First, it calculates the ratio of valid trajectory pixels to the total number of pixels in the image to obtain the trajectory density value. Next, it sets an adaptive threshold based on the median of the trajectory density values in the training set and compares the calculated density with this threshold. Finally, based on the comparison results, the trajectory image is classified into two categories: high density and low density. The extraction mode is automatically switched based on the segmentation results. For high-density trajectory images, a 1 × 1 convolution is first applied to reduce the channel dimension and minimize redundant computations, followed by a 3 × 3 standard convolution to capture the detailed features of local trajectory clusters. For low-density trajectory images, dilated convolution is used to expand the receptive field and capture the global distribution patterns of trajectories.

**Figure 8:**
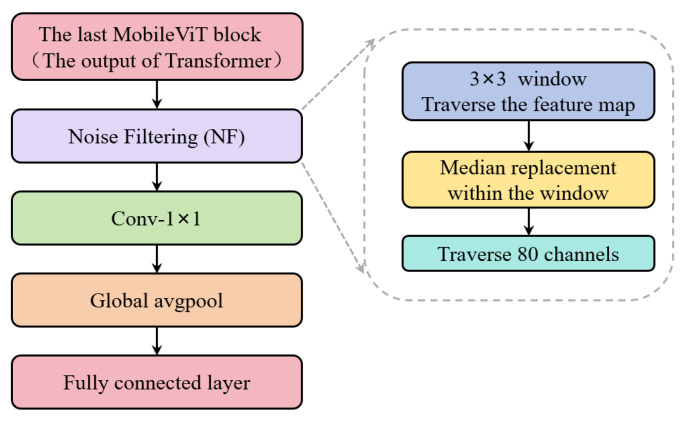
Noise-filtering (NF) module.

The NF module is positioned after the final MobileViT encoding block containing a Transformer layer in the original model, and before the 1 × 1 channel-aggregating convolution and global average pooling. The main structure is shown in Figure 8. In the original model, the global features output by the Transformer encoder are directly fed into subsequent convolutional blocks. Since trajectory images contain spatially isolated noise points, the spatial dimensions of the output features are intermixed with irrelevant interference. This redundancy is amplified after global average pooling, reducing the discriminative power of the final features. This module processes feature maps from all 80 channels sequentially. First, it performs a 3 × 3 window-based pixel-by-pixel scan on the current channel to capture the spatial neighborhood relationships among pixels. Next, it replaces each pixel within each window with its median value, thereby smoothing out discrepancies between isolated noise points and surrounding pixels. This ensures that the trajectory features across all channels undergo spatial refinement, thereby enhancing the spatial coherence of the trajectory regions.

### 3.3. Feature Fusion

**Figure 9:**
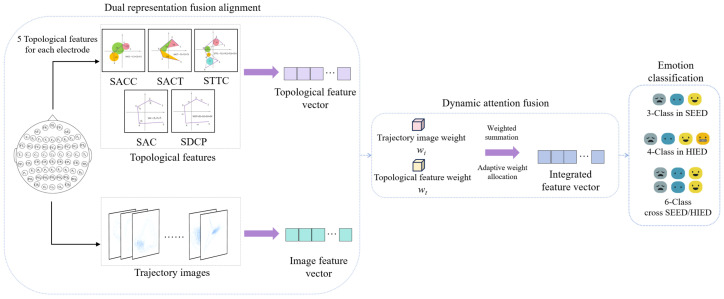
The overall framework diagram of integration.

Unlike conventional multimodal fusion that combines heterogeneous features from unrelated sources, our two representations stem from the same dynamical system, providing a natural basis for complementary integration. Topological features quantify the overall structure of trajectories using metrics such as area, angle, and distance. They can characterize the general dynamic patterns of EEG signals at the macro level and offer strong stability and interpretability. Trajectory images, on the other hand, preserve the specific spatial distribution and local details of trajectories in a plane, reflecting their fine-grained evolutionary characteristics at the micro level. They can be used to automatically extract complex nonlinear patterns through deep learning. These two approaches express information about the same dynamic process from the perspectives of overall structure and local morphology, respectively, and possess complementary advantages. Therefore, a dynamic attention mechanism is introduced to fuse dual representations. By adaptively allocating weights, this mechanism achieves the synergistic integration of macroscopic structural information and microscopic morphological details. After topological features and trajectory images undergo morphological and dimensional alignment, they are subsequently fed into the dynamic attention module for adaptive fusion, ultimately completing emotion classification. The overall framework of the fusion is shown in Figure 9.

The core prerequisite for dual-representation fusion is feature alignment; only when the two types of features are consistent in terms of the objects they describe, their temporal scope, and their semantic referents can feature misalignment be avoided, and effective information complementarity be achieved [29].

**Figure 10:**
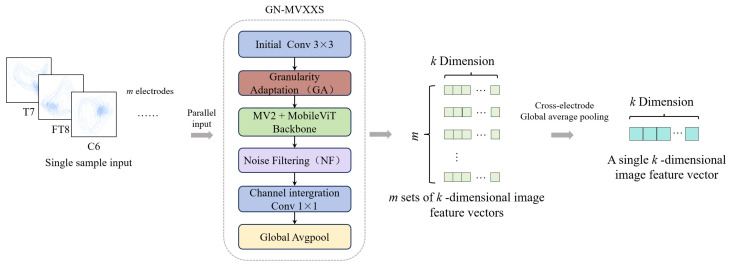
Morphological alignment.

The morphological alignment process is illustrated in Figure 10. First, the trajectory images are fed into the GN-MVXXS model one by one. After each image undergoes trajectory density adaptation via the model’s GA module and feature extraction via the NF module, global average pooling is applied to fully preserve trajectory details and structural information. Subsequently, global average pooling is applied to the image feature vectors, aggregating them into a single vector. This operation integrates the trajectory features from all electrodes, forming a comprehensive representation of the trajectory morphology for a single window sample.

**Figure 11:**
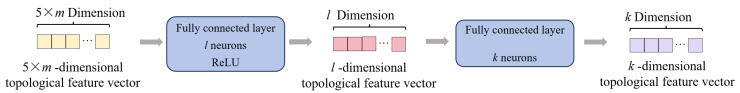
Dimension alignment.

After completing feature alignment, dimensional alignment must be performed. As shown in Figure 11, nonlinear dimensionality expansion is applied to the topological features. A two-layer fully connected network is used for the nonlinear dimensionality expansion, and the ReLU activation function is introduced to enhance the nonlinear expressive power of the features. The second layer fully aligns the expanded features with the dimensionality of the image features. This process preserves the global dynamic information in the topological features that is critical for emotion classification and achieves a distribution match with the image features. Specifically, the image feature vector extracted by GN-MVXXS has 320 dimensions, and the topological feature vector is expanded to 320 dimensions via the two-layer fully connected network.

**Figure 12:**
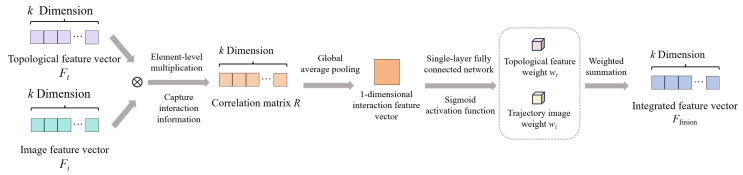
Dual-representation fusion process.

The core of dual-representation fusion lies in achieving effective complementarity; however, a direct concatenation approach does not account for individual differences among samples, which can lead to fluctuations and result in the suppression of useful information. To this end, this study employs a dynamic attention-based fusion strategy based on fully aligned dual-representation feature vectors to achieve efficient information complementarity. The specific fusion method is illustrated in Figure 12.

During fusion, the interaction information is first captured, and the correlation between the topological feature vector $F_{t}$ and the image feature vector $F_{i}$ is modeled. The element-wise correlation matrix R between the two representations is then calculated, as shown in the following equation:

(20) $$R=F_{t}⊗F_{i}$$

where ⊗ represents element-wise multiplication, which can capture the interaction between these two types of features.

Next, global average pooling is applied to the correlation matrix R to obtain a one-dimensional interaction feature vector representing the degree of synergy between the two representations. After passing through a single-layer fully connected network with a sigmoid activation function, the model outputs the attention weights $w_{t}(w_{t}\in [0,1])$ for topological features. According to the principle of complementarity, the sum of the weights for the two representations is one; thus, the attention weights $w_{i}=1-w_{t}$ for image features are determined.

Finally, the two feature representations are fused using a weighted summation to obtain the final fused feature $F_{\mathrm{fusion}}$, as shown in the following formula:

(21) $$F_{\mathrm{fusion}}=w_{t}\times F_{t}+w_{i}\times F_{i}$$

$w_{t}$ and $w_{i}$ vary dynamically with the sample; for samples with significantly different trajectory topologies, $w_{t}$ automatically enhances the prominence of image features; for samples with higher resolution in trajectory details, $w_{i}$ automatically enhances them to highlight the prominence of image features.

## 4. Experimental Results

### 4.1. Experimental Setup

For the SEED, relevant clips were selected to conduct subject-dependent experiments for binary classification (happy, sad) and ternary classification (happy, neutral, sad). For the HIED, relevant clips were similarly selected to conduct subject-dependent experiments for binary classification (happy, sad), ternary classification (happy, neutral, sad), and quadruple classification (happy, neutral, sad, fearful). All experiments utilized the first 80% of the clip samples as the training set and the remaining 20% as the test set. This approach prevents different instances of the same emotional movie clip from being assigned to both the training and test sets simultaneously, thereby avoiding data leakage. For subject-dependent experiments, no separate validation set or cross-validation was applied; the reported accuracies are based on the fixed 80/20 split. For the classification task across normal-hearing and hearing-impaired participants, the selected categories were sad, neutral, and happy from SEED and the same three emotions from HIED. A leave-one-subject cross-validation scheme was employed, where each of the 30 subjects was used once as the test set while the remaining 29 served as the training set. The experiments were run using the PyTorch deep learning framework with Python version 3.9, and the software used was PyCharm 2020.1.1 x64. Model training was accelerated using a GPU, specifically an NVIDIA GeForce RTX 3060 12G. The model is optimized using the cross-entropy loss function for multi-class classification.

### 4.2. Selection of EEG Channels

**Figure 13:**
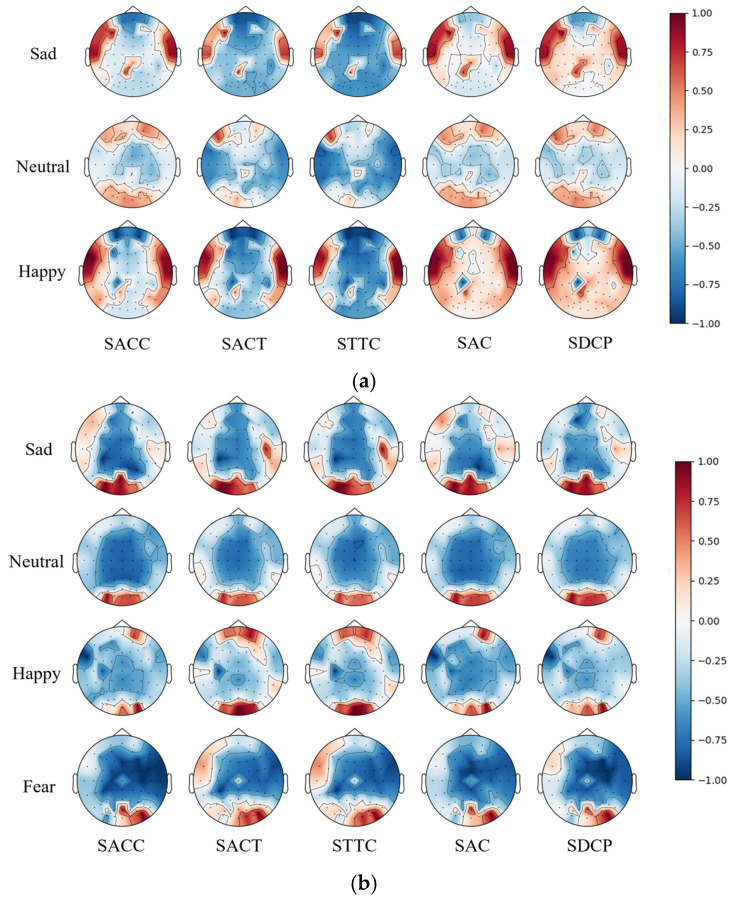
Average brain topographic maps of five topological features across different emotions. (**a**) Normal-hearing participants in the SEED. (**b**) Hearing impairments in the HIED.

To reduce the computational complexity of integrating topological and trajectory features into the model, we plotted and analyzed the brain maps of the five selected topological features based on the normalized average samples from hearing and hearing-impaired participants across different emotions. As shown in Figure 13, in normal-hearing individuals, the significant brain regions associated with neutral emotions are concentrated in the frontal and occipital lobes. Compared to neutral emotions, sadness and happiness produced varying degrees of activation in the temporal lobe. In hearing-impaired individuals, activation in the occipital lobe was more pronounced, while activity in the temporal lobe was significantly lower. This may be related to visual compensation mechanisms during visual processing in the brain; the absence of auditory input promotes compensatory enhancement of visual and somatosensory pathways and, through cross-modal neural reorganization to optimize resource allocation, leads to improved processing efficiency of emotion-related visual cues in the occipital lobe.

**Figure 14:**
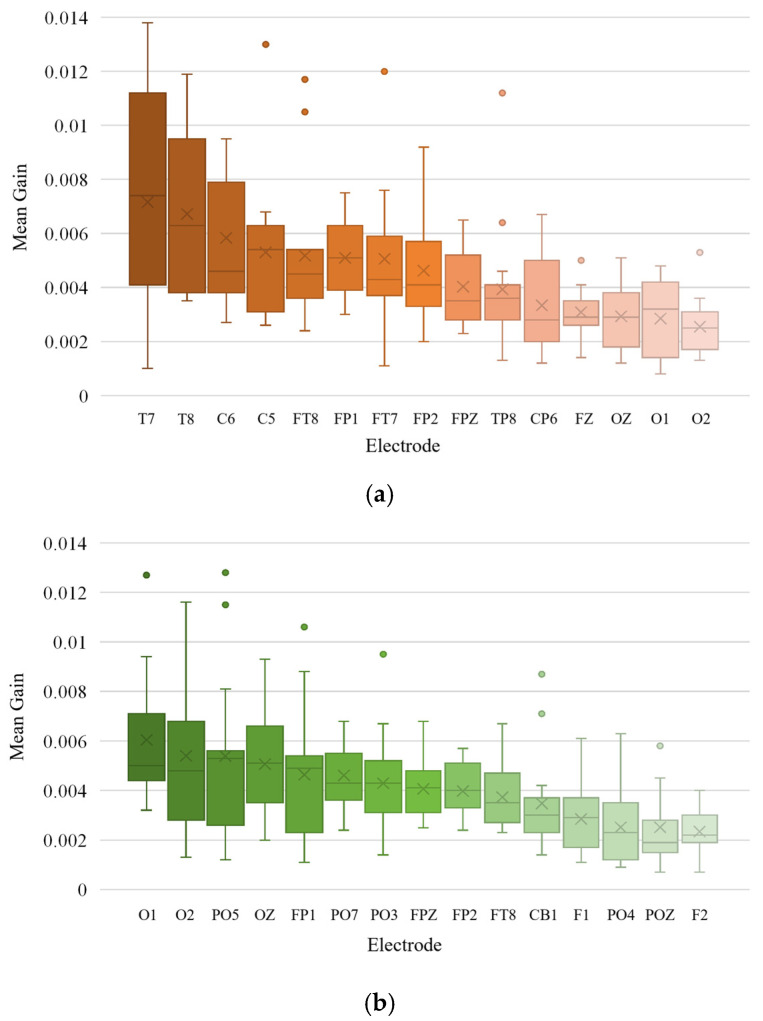
XGBoost: Top-ranked feature channels by gain (in descending order). (**a**) Normal-hearing participants in the SEED. (**b**) Hearing impairments in the HIED.

**Figure 15:**
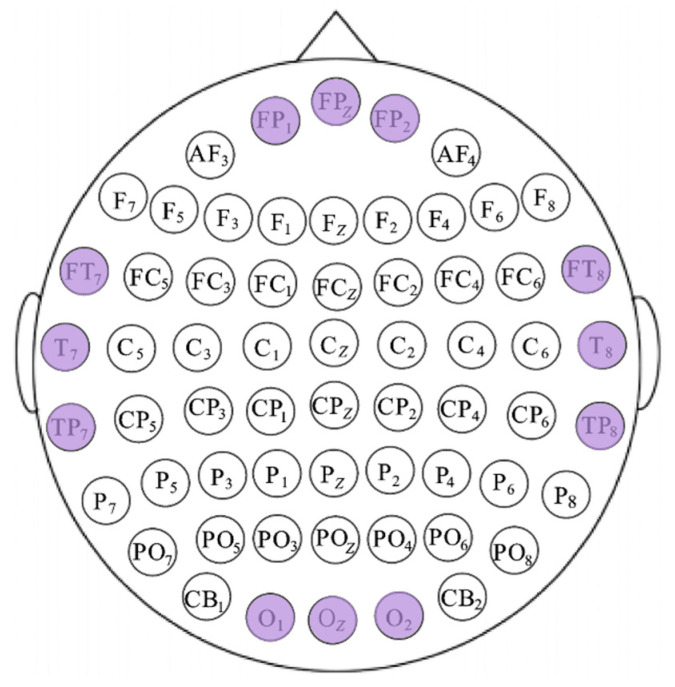
Schematic diagram of electrode selection, with the purple sections indicating the selected electrodes.

In addition, we calculated the average XGBoost gain for each feature in every electrode channel and plotted a box plot of the highest-ranked average channel gains in descending order, as shown in Figure 14. In normal-hearing individuals, electrodes with higher gains are primarily concentrated in the temporal lobe region (T7, T8, C6, C5, FT8, FT7, TP8, CP6). In addition, the parietal lobe region (FP1, FP2, FPZ, FZ) and the occipital lobe region (OZ, O1, O2) also make significant contributions. Among hearing-impaired participants, the number of high-gain electrodes in the occipital lobe region was significantly higher than in hearing-impaired participants (O1, O2, PO5, OZ, PO7, PO3, CB1, PO4, POZ), while the temporal lobe included only FT8. This indicates that emotion classification in hearing-impaired participants relies more heavily on the occipital lobe, which is consistent with the previous discussion. The remaining electrodes with significant contributions are primarily located in the parietal lobe (FP1, FPZ, FP2, F1, F2). Based on the above findings, and by combining the distribution of key brain regions across the two groups of participants while considering common regions and bilateral symmetry, 12 high-gain electrodes were selected, as shown in Figure 15.

### 4.3. Feature Fusion Results

Trajectory image data were acquired using 9 s non-overlapping time windows that were identical to those used for topological feature extraction, ensuring complete consistency in sample segmentation. Additionally, based on the *m* = 12 selected key electrode channels, each channel corresponds to a separate trajectory image, enabling the visualization and independent characterization of electrode signals. The total number of image samples is calculated as “number of windows × number of electrode channels × number of video clips”. In the SEED, the total number of image samples per subject is 20 × 12 × 15 = 3600; in the HIED, the total number of image samples per subject is 20 × 12 × 20 = 4800.

**Figure 16:**
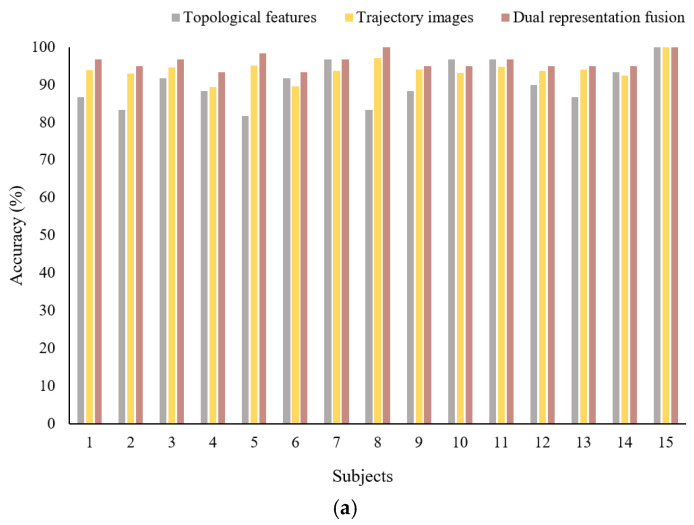

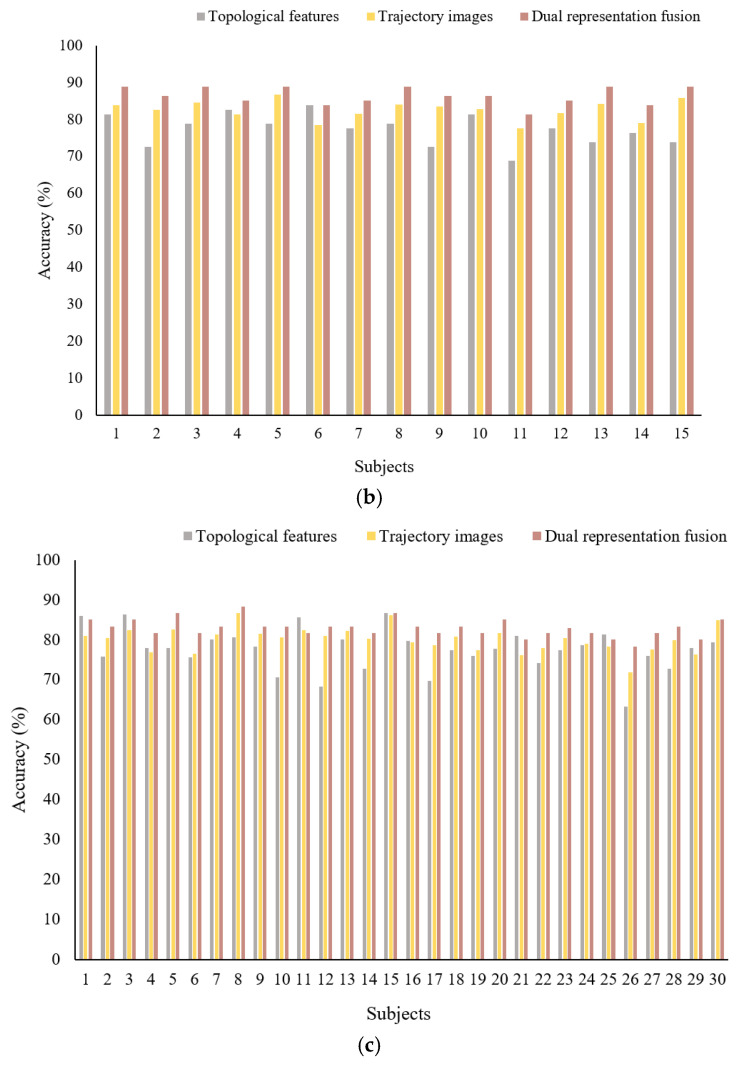
Classification results from dual-representation fusion based on the GN-MVXXS model. (**a**) Three-class classification of the SEED. (**b**) Four-class classification of the HIED. (**c**) Classification across normal-hearing and hearing-impaired people (Subjects 1–15: normal hearing; Subjects 16–30: impaired hearing).

As shown in Figure 16, the dual-representation fusion framework demonstrates significant performance gains across all three task types. In the three-class classification task on the SEED, the average accuracy achieved by the dynamic attention fusion method reached 96.11%, representing an improvement of 5.78% over topological feature recognition and 2.24% over trajectory image recognition, highlighting the complementary value of dual-representation information. In the four-class classification task on the HIED, the accuracy of the dynamic attention fusion method was 86.33%, representing improvements of 9.16% and 3.84% over topological feature recognition and trajectory image recognition, respectively. Due to visual compensation mechanisms, the trajectory characteristics of hearing-impaired individuals are more distinctive, and the limitations of single-representation methods are more pronounced; however, dual-representation fusion effectively integrates global dynamic patterns with local morphological details, mitigating this limitation. In the classification task across normal-hearing and hearing-impaired individuals, the dynamic attention fusion achieved an average accuracy of 83.67%, which was also higher than the results obtained from topological feature recognition and trajectory image recognition. This indicates that the fusion framework not only improves recognition performance within a single group but also enhances generalization capabilities in cross-group scenarios.

To evaluate the performance of the dynamic attention fusion approach, we compared it with the direct concatenation method across three tasks; the results are shown in Table 1. As can be seen, dynamic attention fusion demonstrates a significant advantage in all tasks, with an average accuracy that is consistently higher than that of direct concatenation. This demonstrates that by modeling the interaction between dual representations to adaptively assign weights, the approach can precisely achieve complementary information exchange, thereby overcoming the limitations of fixed weights in direct concatenation and fully validating its stability.

## 5. Discussion

### 5.1. Confusion Matrix

**Figure 17:**
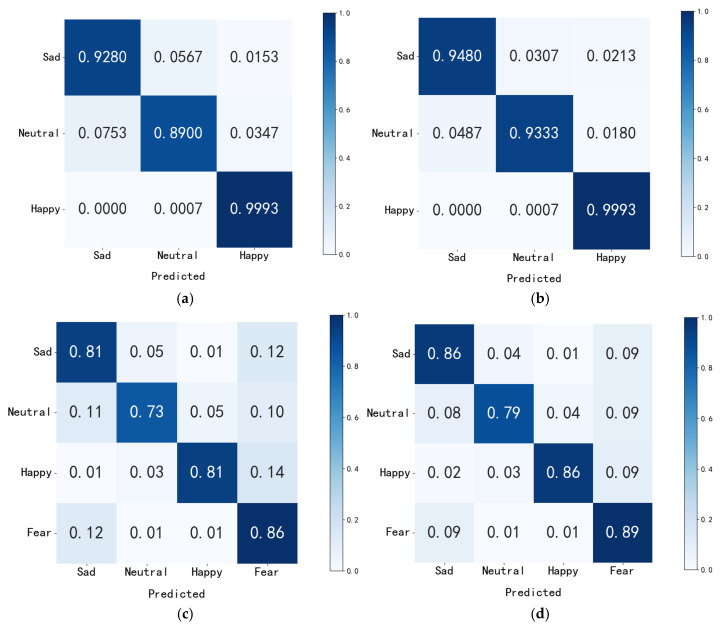

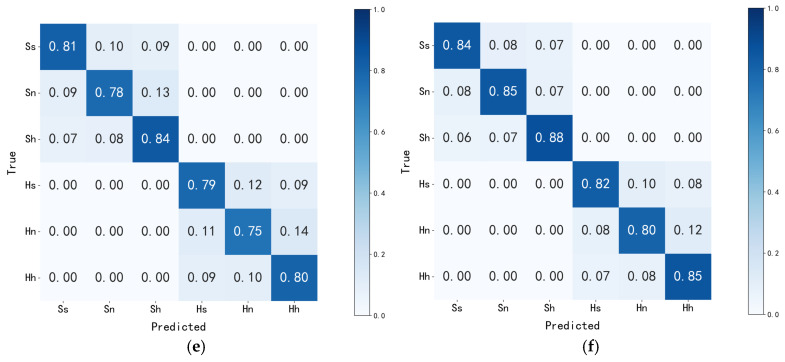
Confusion matrices for different fusion schemes across various tasks. (**a**) Direct concatenation of the three SEED categories (**b**) SEED Three-category dynamic attention fusion. (**c**) Direct concatenation of the HIED four-classification. (**d**) HIED four-class dynamic attention fusion. (**e**) Direct concatenation (across normal-hearing and hearing-impaired people). (**f**) Dynamic attention-based fusion (across normal-hearing and hearing-impaired people).

To verify the discriminative effectiveness of dynamic attention fusion compared to direct concatenation fusion, we conducted an analysis using confusion matrices for the three-class task, as shown in Figure 17. In the three-class classification task on the SEED, dynamic attention fusion effectively mitigated the confusion issue regarding neutral emotions. While approximately 7% of neutral samples were misclassified as sad in the direct concatenation approach, dynamic attention fusion significantly reduced this misclassification rate by enhancing the subtle differences in image features.

In the four-class classification task on the HIED, dynamic attention fusion resulted in significant improvements in recognition performance across all emotion categories compared to direct concatenation. This demonstrates that dynamic attention fusion accurately captures the unique representations of each emotion category by deeply exploring the global dynamic patterns of topological features and the local details of trajectory images. At the same time, it accommodates the unique trajectory characteristics of individuals with hearing impairments resulting from visual compensation mechanisms, leading to a more balanced distribution of classifications across emotion categories. In the classification task across normal-hearing and hearing-impaired participants, dynamic attention fusion further enhances the suppression of group-specific interference and the effective focusing of features by modeling the interactive correlations between dual representations and group-common emotional representations. This approach achieves a significant improvement in the average accuracy of intra-group emotion recognition, demonstrating the value of this fusion strategy.

### 5.2. Dynamic Weight Selection

**Figure 18:**
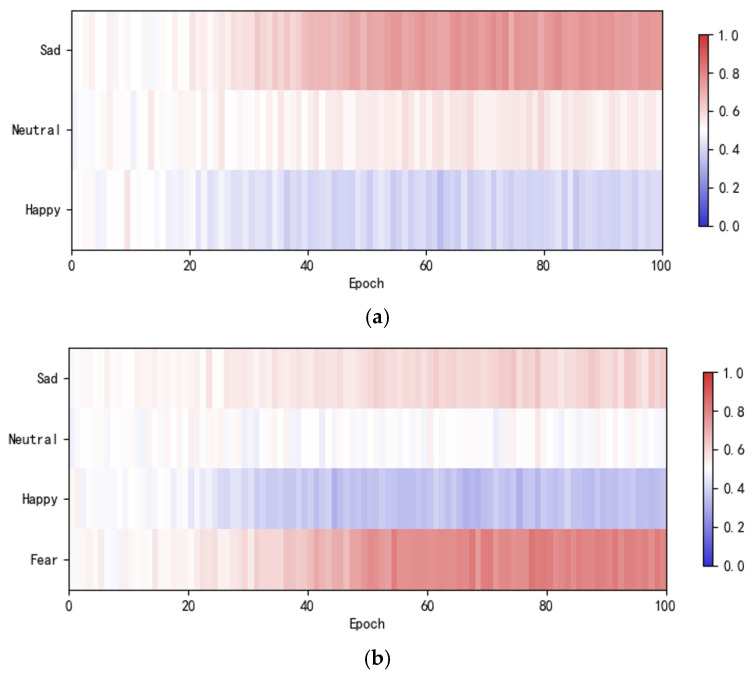

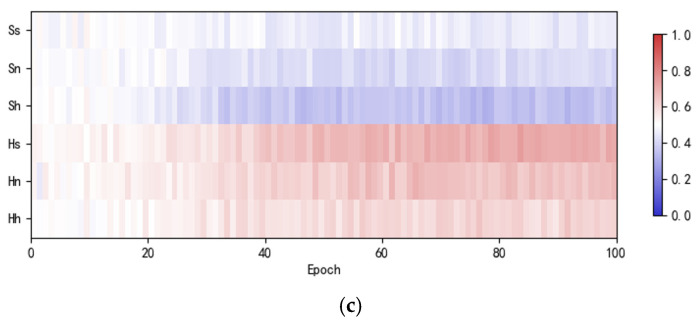
Dynamic changes in weights during training for different tasks. (**a**) Three-class classification of the SEED. (**b**) Four-class classification of the HIED. (**c**) Six-class classification (across normal-hearing and hearing-impaired people).

Figure 18 illustrates the dynamic changes in weights across different sentiment categories during training (deeper red indicates stronger dominance of topological features, while deeper blue indicates stronger dominance of trajectory images). In the initial stage, the weights for each emotion are close to 0.5 (white region), indicating that the model has no significant bias toward either representation. As training iterations progress, the weights gradually diverge. This evolutionary process intuitively demonstrates that the dynamic attention mechanism can adaptively adjust representation dependencies based on task characteristics, achieving a learning process that transitions from initial equilibrium to final divergence.

**Figure 19:**
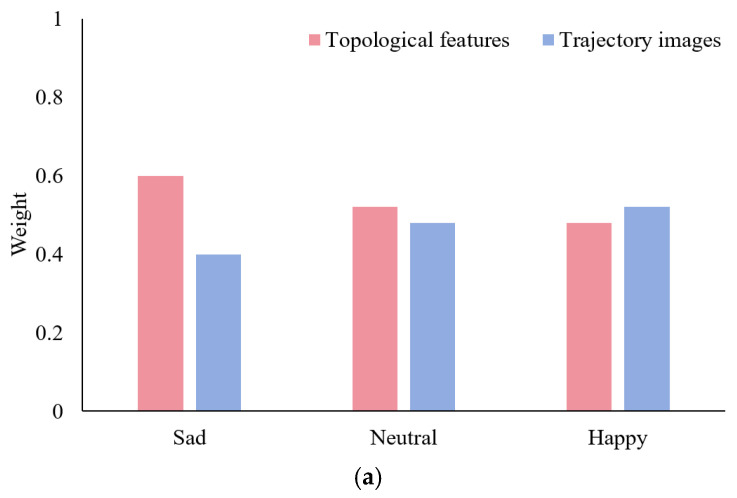

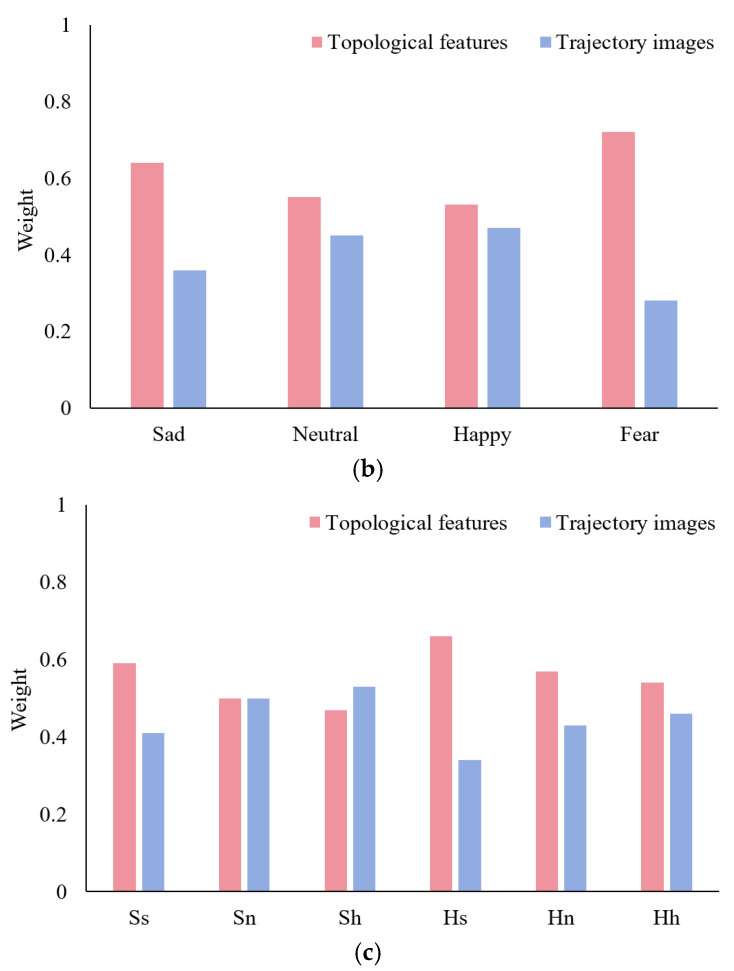
Dual-representation weights for each sentiment category in the test set under the same task. (**a**) Three-class classification of the SEED. (**b**) Four-class classification of the HIED. (**c**) Six-class classification (across normal-hearing and hearing-impaired people).

Figure 19 summarizes the final average weights of each emotion category on the test set, providing a visual representation of the differences in how various emotions rely on dual representations. In the SEED three-class classification, sadness showed the highest dependence on topological features (0.60), happiness showed the lowest (0.48), and neutral emotions fell in the middle (0.52); in the HIED four-class classification, fear exhibited a significantly higher weight for topological features than other emotions (0.72), happiness showed the lowest (0.53), and sadness (0.64) and neutral emotions (0.55) fell in the middle. In the cross-group six-class classification, the topological feature weights for emotions in the hearing-impaired group were all higher than those for the corresponding emotions in the normal-hearing group. The above distributions are highly consistent with the evolutionary endpoints shown in Figure 18, validating that the dynamic fusion strategy can adaptively allocate representation contributions based on the characteristics of different emotions and groups, thereby achieving complementary advantages.

### 5.3. Comparison with Existing Research

Table 2 presents the results of our various experiments, as well as a comparison with existing studies on the SEED. The topological features proposed in this paper effectively capture the nonlinear neural activity patterns associated with emotional states. The GN-MVXXS model can deeply extract emotion-related features from trajectory images, while the dynamic attention mechanism seamlessly integrates these two components, achieving competitive recognition performance on the SEED, though slightly lower than some recent specialized methods. Nevertheless, our framework offers unique advantages in cross-dataset generalization and, more specifically, in generalization across normal-hearing and hearing-impaired populations.

In order to distinguish the contribution of feature representation from that of the classifier architecture, we fixed the classifier to XGBoost and only changed the input features. The results showed that the concatenated features (91.54%) were superior to the two single-feature baselines (90.33% and 88.65%), demonstrating the inherent complementarity of these two representations. The complete model (96.11%) was further 4.57% higher than this concatenated baseline, which was attributed to the dynamic attention fusion mechanism.

## Figures and Tables

**Table 1:** Comparison of experimental results across three tasks.

Method	SEED Tasks	HIED Tasks	Cross-Group Tasks
Direct Splicing (%)	94.44	82.75	79.28
Dynamic Attention Fusion (%)	96.11	86.33	83.67

**Table 2:** The results of our various experiments, as well as the comparison with existing studies on the SEED.

Method	Features/Modalities	Classifier	Accuracy
Xu et al. [29]	HOC, FD, band power, DE	SVM	81.90%
Yan et al. [30]	DE, PSD, DASM, RASM, DCAU	Spatio-temporal Graph Bert network	83.20%
Kouti et al. [31]	iCoh connection feature	SVM	83.84%
Sun et al. [32]	RMS + DE	RF	88.93%
Kumar et al. [33]	DE	BiLSTM	93.05%
Kotwal et al. [34]	DE	CNN	94.09%
Li et al. [35]	DE	DenseNet	96.73%
Esmi et al. [36]	2D spatio-temporal–spectral image features	TEREE	97.70%
Ours	Topological features	XGBoost	90.30%
Ours	Trajectory image	XGBoost	88.65%
Ours	Topological features and trajectory plots	XGBoost	91.54%
Ours	Trajectory image	GN-MVXXS	93.87%
Ours	Topological features and trajectory plots	Direct Splicing	94.44%
Ours	Topological features and trajectory plots	Dynamic Attention Fusion Network	96.11%

## Data Availability

The data is unavailable due to privacy or ethical restrictions.
